# Global Monocular Indoor Positioning of a Robotic Vehicle with a Floorplan †

**DOI:** 10.3390/s19030634

**Published:** 2019-02-02

**Authors:** John Noonan, Hector Rotstein, Amir Geva, Ehud Rivlin

**Affiliations:** 1 Department of Computer Science, Technion—Israel Institute of Technology, Haifa 3200003, Israel; amirgeva@cs.technion.ac.il (A.G.); ehudr@cs.technion.ac.il (E.R.); 2 Department of Electrical Engineering, Technion—Israel Institute of Technology, Haifa 3200003, Israel; hector@ee.technion.ac.il

**Keywords:** indoor positioning, robotic vehicle, vision-based navigation, floorplan

## Abstract

This paper presents a global monocular indoor positioning system for a robotic vehicle starting from a known pose. The proposed system does not depend on a dense 3D map, require prior environment exploration or installation, or rely on the scene remaining the same, photometrically or geometrically. The approach presents a new way of providing global positioning relying on the sparse knowledge of the building floorplan by utilizing special algorithms to resolve the unknown scale through wall–plane association. This *Wall Plane Fusion* algorithm presented finds correspondences between walls of the floorplan and planar structures present in the 3D point cloud. In order to extract planes from point clouds that contain scale ambiguity, the *Scale Invariant Planar RANSAC* (SIPR) algorithm was developed. The best wall–plane correspondence is used as an external constraint to a custom Bundle Adjustment optimization which refines the motion estimation solution and enforces a global scale solution. A necessary condition is that only *one* wall needs to be in view. The feasibility of using the algorithms is tested with synthetic and real-world data; extensive testing is performed in an indoor simulation environment using the *Unreal Engine* and *Microsoft Airsim*. The system performs consistently across all three types of data. The tests presented in this paper show that the standard deviation of the error did not exceed 6 cm.

## 1. Introduction

Global localization for robotic vehicles is an essential backbone for robust autonomous navigation. In outdoor applications, the Global Positioning System (GPS) can be used to compute an accurate and inexpensive position solution, which unfortunately is either not available or substantially degraded in indoor environments due to the blocking of signals by the building structure. For this reason, the indoor localization problem is still unsolved in general and attracts considerable research efforts both in academia and in industry.

In determining the system to use for positioning, it is important to consider the extent of applications that would utilize it. Envisioned applications range from autonomous inspection of hazardous factories to autonomous exploration of unknown, volatile compounds to emergency medical delivery for victims trapped in buildings and even to product transportation and distribution within large warehouses. For such situations, having a system which does not depend on prior environment setup or exploration is vital. Furthermore, the question of system setup prior to execution becomes a prime concern when handling emergency situations. For example, while ultra-wideband (UWB) positioning [1] has been often considered as a possible alternative, the requirement of a priori infrastructure installation or deployable equipment prevents it from being a feasible solution in the context of the present study.

In this paper, a vision-based approach to the positioning problem is proposed using the images acquired by a monocular camera. The use of a single camera is attractive due to the inherent low costs and ease of integration into almost any robotic vehicle. On the other hand, monocular vision by itself provides motion information up to scale, and hence estimation of global positioning requires resolving scale in an effective manner. The approach pursued in this paper is to complement the visual information acquired by a monocular camera with the knowledge of the building floorplan. The latter is easily accessible for many indoor environments and requires no prior equipment installation or exploration. Moreover, as shown here, it can be used to resolve scale through wall–plane association and thus provide monocular global localization within the building of interest. The new approach only uses the planar information of the floorplan (normal vectors and distance values according to a predefined global coordinate system), which is actually only a subset of the information provided by a floorplan. Although based on looking at features on walls, one needs to consider that objects other than walls may abound in indoor environments; these objects are classified as *obstacles*. For reasons to become clear below, obstacles may be classified as *non-planar* such as chairs, desks or tables, or *planar* such as bookshelves, cabinets or boxes. In either case, any relevant vision-based positioning system using a floorplan to achieve absolute localization must be able to filter these obstacles out at a pre-processing stage. A preliminary, shorter version of the paper was presented at the *2018 International Conference on Indoor Positioning and Indoor Navigation* [2]. This manuscript extends the conference paper in the following new ways:

- *Unreal Engine & Microsoft Airsim* Indoor Simulation testing (Section 8),
- Positioning update routine following initialization (Section 5),
- Concavity Filter in the *Wall Plane Fusion* (WPF) algorithm (Section 4.4),
- New score functions for the WPF best wall–plane pair computation (Section 4.4),
- Additional cost terms for the constrained Bundle Adjustment optimization (Section 6),
- Real data test comparison with a method combining ORB-SLAM2 with LIBVISO2 (Section 8),
- Computational runtime discussion (Section 7),
- Proof of the Planar Extraction Lemma (Appendix B),
- *All new* experimentation with the modified positioning system yielding more accurate results (Section 8),
- More in-depth analysis of the entire indoor positioning system.

The robotic platform in which the new positioning systems were tested is the BARC (see http://www.barc-project.com/), a 1/10th scale 4-wheel vehicle augmented with a monocular camera and on-board wheel encoders. The focus is to utilize the floorplan along with special algorithms to resolve the unknown scale and provide global positioning information. The BARC is especially challenging for this kind of approach as compared with, e.g., the research presented in [3] which considers the similar localization problem of a quadcopter inside a building using a monocular camera and the floorplan. As explained here, the low profile of the ground vehicle considered makes the practical implementation substantially more challenging. This is highlighted, in particular, by the attention required for the wall association and obstacle detection problems that were not considered in [3].

**Figure 1:**
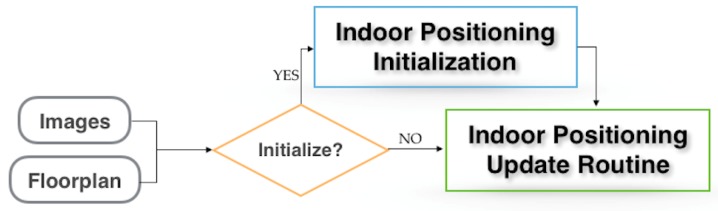
Positioning system flow diagram.

The positioning algorithm flow diagram is shown in Figure 1. The figure shows that the input data consists of a collection of images taken by the moving robot and the floorplan of the building it is moving in. The algorithm consists of two main blocks: (1) Initialization and (2) Update. In the initialization block, the scale for the global motion of the vehicle is established by recognizing and exploiting planar structure in the sparse point cloud with a wall of the floorplan. In the Update block, the algorithm continually uses images of the environment while the robot is moving to compare with the floorplan, thus providing global positioning through wall-point mappings.

The rest of the paper is organized as follows: Section 2 discusses current related work in the area of monocular vision localization; Section 3 provides a simple example of the entire positioning system; Section 4 provides details about the initialization framework and its design; Section 5 presents how the system produces continual positioning updates; Section 6 describes how a wall–plane association can be exploited as an external constraint to refine the camera motion via Bundle Adjustment optimization and enforce a global scale solution; Section 7 discusses the computational runtime of the system; Section 8 provides results running synthetic, simulation, and real-world tests on the localization system; and Section 9 concludes the paper.

## 2. Related Work

Monocular Simultaneous Localization and Mapping (SLAM) has been a targeted research topic recently, mainly due to its wide-ranging potential applications. By itself, SLAM estimates the relative camera motion while at the same time reconstructing the environment in which the camera moves. Approaches can be categorized as either *feature-based* methods such as ORB-SLAM2 [4] where features (e.g., ORB) are extracted from images to be used for tracking and the error minimized is based on the 2D feature positions, or *direct* methods such as Direct Sparse Odometry (DSO) [5] which is featureless, and the error is based on pixel intensities. Monocular SLAM is also sometimes coupled with an inertial system, giving rise to integrated visual-inertial SLAM [6,7,8,9].

Given that arguably the main challenge when using a monocular camera is the ambiguity in scale or depth of the scene, researchers have investigated various ways of resolving scale so that global monocular localization or “visual odometry” can be achieved. It is important to note that, in order for visual odometry to be applied, the initial camera pose needs to be known. One approach is called object-SLAM and its goal is to recognize objects in the environment and compute the scale utilizing priors on the sizes of such objects [10,11,12,13]. Alternatively, some methods rely on the fact that the camera moves over a planar surface with constant altitude. Thus, the goal becomes endeavoring to detect the road/floor region and estimate the ground plane or geometrical structure to resolve the scale [14,15,16,17]. In addition, other works have presented systems to learn the scale via convolutional neural networks [18,19,20,21]. The proposed positioning system alleviates some of the challenges inherent to the aforementioned approaches. For example, in view of the ground plane estimation approach, it is often the case that feature points on walls are more prevalent than feature points on the floor. Thus, the proposed system relies on such features. In addition, using a floorplan which is architecturally measured provides much better and more accurate a priori information than object size priors.

Feng et al. [22] approached global monocular localization utilizing a pre-built, dense 3D map of an indoor environment, consisting of database images with respective global camera poses and with each pixel mapped to a 3D world point. Although in principle attractive, this approach is far from being ideal. An indirect assumption is that from the time the 3D map is created to the time of running the localization system, the scene has not photometrically nor physically changed in a significant manner. Furthermore, relying on a dense 3D map with prepared database images means that the environment needs to be explored prior to the localization, which is not always desirable or possible. Caselitz et al. [23] developed a monocular localization system using a priori information of a 3D LiDAR map. While such an approach alleviates photometric issues since it is a geometric correspondence problem, it still requires pre-visiting the environment to create the map. In addition, it assumes no geometric changes to the scene are made before localization, for example by introducing new obstacles or by rearranging current obstacles in the environment. For the case considered in this paper, using a floorplan neither requires traversing the scene before localization nor depends on a dense 3D map. Moreover, the algorithm is insensitive to changes in the scene: obstacles can be introduced or adjusted without significantly affecting localization results as long as the floorplan itself is not modified.

## 3. Simple Example of the Positioning System

**Figure 2:**
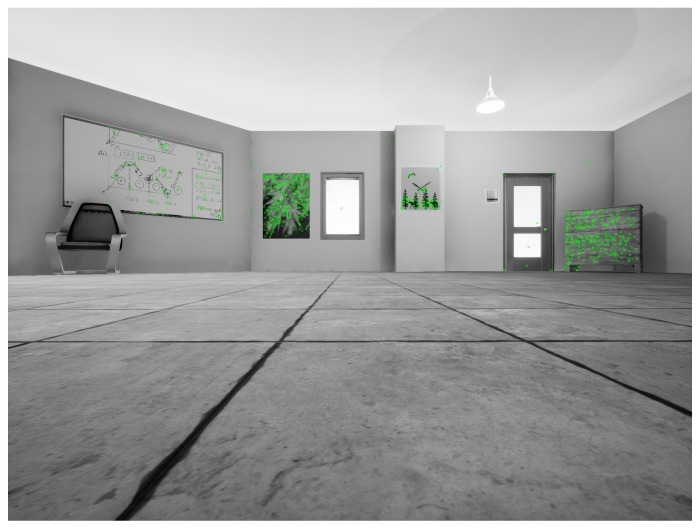
View of an environment with computed features.

This section serves to provide intuition for how the system runs by providing a simple example. Subsequent sections delve into the details of each of the system’s modules. The algorithm first extracts features from the batch of images and computes tracks across the frames (Figure 2).

**Figure 3:**
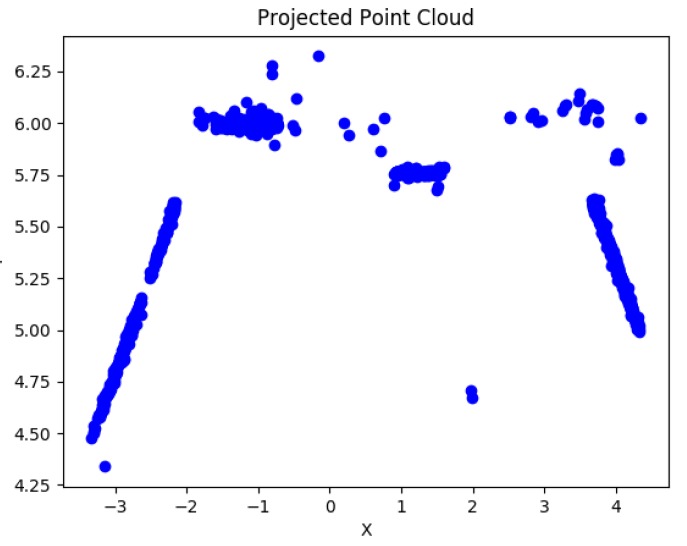
Top view projection of the unscaled point cloud from SfM.

An initial estimate of the 3D geometry of the environment and the camera motion up to scale is obtained by running a general Structure from Motion (SfM) algorithm (Figure 3).

**Figure 4:**
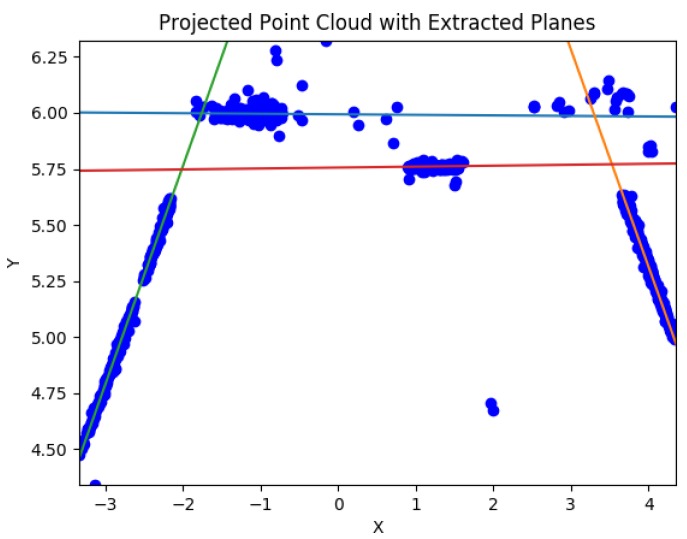
Extracted planes from the point cloud.

The point cloud computed by SfM is subsequently passed to the new *Scale Invariant Planar RANSAC* (SIPR) algorithm, developed to extract the underlying planar structures of the scene; it is called **scale invariant** because it is able to extract planes from point cloud data with scale ambiguity (Figure 4).

**Figure 5:**
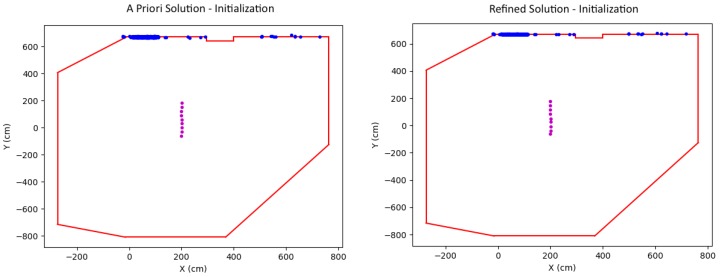
Initialization: scaled camera motion and world points before (**left**) and after (**right**) optimization.

Subsequently, scale is recovered using the *Wall Plane Fusion* (WPF) algorithm discussed below, which finds correspondences between the computed planes and the walls as defined by the floorplan. In order to use the WPF, one needs to deal with both planar and non-planar obstacles present in all typical scenes and not included in the floorplan. As explained next, non-planar obstacles are handled in the SIPR algorithm, while planar obstacles are filtered out by the WPF algorithm. The final output is a best estimate for the wall–plane pair, representing the computed plane which most closely matches a wall of the floorplan in view *and* the resulting alignment scale estimate. Figure 5 (left) shows the best wall that was selected with the active 3D points on it after they were properly scaled according to the scale estimate. A specially designed, constrained Bundle Adjustment optimization is run exploiting this best wall–plane pair as an external constraint, returning a global positioning solution and refined 3D geometry (Figure 5 right). Note that a perfect line is not what is expected since the ground truth should have a path which is not exactly a straight line.

**Figure 6:**
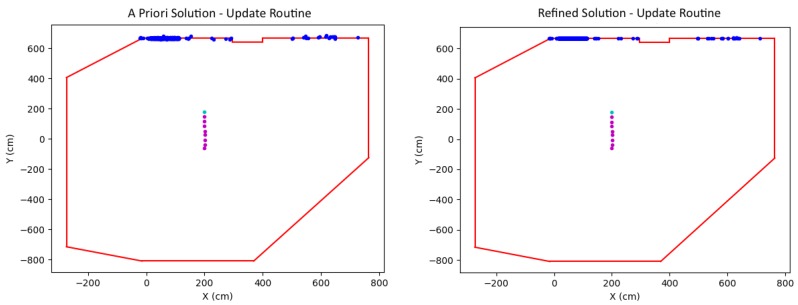
Update routine: new camera position (cyan) and world points before (**left**) and after (**right**) optimization.

After performing initialization, an update routine takes over to handle all subsequent localization. Utilizing the scale computed during initialization, each new camera pose as well as the corresponding 3D points computed from the batch of images are refined in the wall-constrained Bundle Adjustment optimization. Figure 6 shows the solution (camera position and points) before (left) and after (right) the optimization. Note that the illustrations are top view perspectives, and the camera at the new time instance is highlighted in cyan.

## 4. Initialization Module

**Figure 7:**
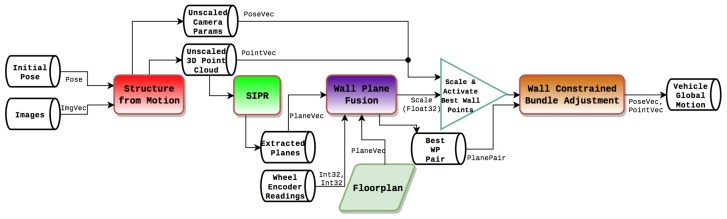
Positioning initialization pipeline.

**Figure 8:**
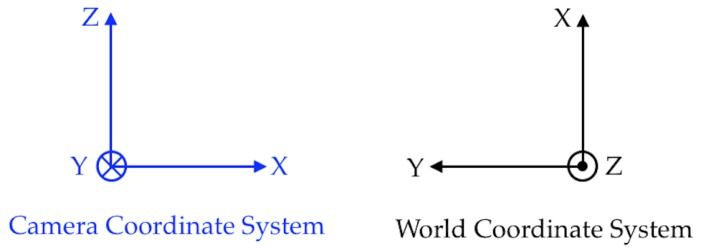
Coordinate systems—Top View; see [2].

The initialization pipeline is shown in Figure 7 and the relevant coordinate systems are shown in Figure 8.

### 4.1. Feature Detection and Tracking

Given a batch of images, feature points were extracted using the SIFT keypoint detector and tracked across multiple frames. In this system, a track was considered valid if the corresponding feature point was seen in at least three frames. For the purposes of this system, the feature tracking aspect was not the focus, and was thus treated more or less in a black-box fashion, where different parameters (e.g., nearest-neighbor ratio, minimum track length, etc.) varied across experiments.

### 4.2. Structure from Motion Stage

Let us assume that at least two images are taken while the robot is performing a motion. Tracked feature points are passed to a multi-frame Structure from Motion algorithm to generate the initial motion and provide a sparse description of the environment (tens to hundreds of features per frame). Note that the motion and the location of the points in the environment are up to scale, but still, as explained next, can be used to extract a number of planes that serve as wall-candidates.

### 4.3. The Scale-Invariant Planar RANSAC

Once a point cloud is calculated, it could in principle be used to compute a number of planes that could be identified with the walls described in the floorplan. However, upon further consideration, a standard algorithm would not perform correctly due to the lack of scale in the data and the fact that all surfaces of interest are in fact planar. Consequently, a new *Scale Invariant Planar RANSAC* (SIPR) algorithm was developed to extract the underlying planar structures of the scene. Once the algorithm extracts planes from point cloud data with scale ambiguity, a good scale estimate can be found by comparing the result with the floorplan. In order for the planar extraction to be accomplished, traditional methods were avoided—methods using predefined thresholds for determining whether points are considered inliers or outliers and for determining the stopping criterion, when all planes in the point cloud have been extracted. The algorithm is described in the following sections.

#### 4.3.1. Plane Initialization

**Figure 9:**
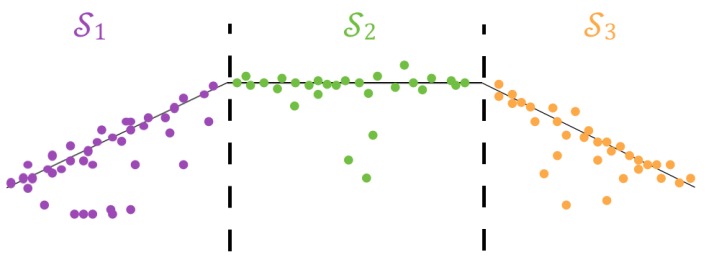
Illustration of the point cloud subdivision for plane initialization.

After transforming the solution to be in the coordinate frame of the camera at the first instance, the point cloud is projected onto the plane parallel to the motion of the vehicle (the xy plane in world coordinates). Because at least three points are needed to form a plane, the projected point cloud is subdivided into three equal subsets. The direction of subdivision is either along the *x*- or *y*-axis depending on which has a higher point distribution range. An illustration of this is shown in Figure 9. Note that, while in the illustration the subdivisions align with the wall boundaries, this is not a necessary condition.

While a standard approach suggests choosing the three points at random with uniform independent probability, this method endeavors to improve the selections. While the first point is chosen with equal probability from each of the subsets, the second point is selected with a higher probability of it being in the same subset as the first point and a lower probability of it being in an adjacent subset. Note that, given a point on a wall, it follows that points nearby have a relatively high likelihood of also being on the same wall. Furthermore, no point is selected from any subset which is not directly adjacent to the first subset. The majority of the time walls will exist in a single subset or span across two adjacent subsets. If a wall exists in all three subsets, then picking from two subsets is sufficient to form the plane. In the case that a wall has a protrusion and that protrusion appears in the second subset, whereas the setback wall occurs in the first and third subsets, choosing only points from either the first or third subset to form the plane is also sufficient for properly finding the plane. At a later stage as a part of the RANSAC process, where all points closest to the plane are collected, both subsets’ points would be included. The third point is chosen similarly to the second point but given an even higher probability of being selected in the same subset as the previous two. Refer to Appendix A for details on the probabilities to compute the initial plane estimate.

#### 4.3.2. The Refined Plane Estimate

After finding an initial plane estimate from three points of the point cloud, the distance error is computed from each point to the plane estimate. Let $e_{max}$ refer to the largest such error. Then, all of the points, $p_{i}$ for i∈1…N, used during this refinement step are placed into an error histogram, $H_{e},$ of size $N_{bins}$, where each bin corresponds to a fraction of the maximum error distance, $e_{max}$. Note that beforehand an initial filtering is done to remove any extreme outliers. Subsequently, the plane estimate is iteratively refined by considering only points in the first bin.

In order to compute the refined plane, note that if the initial plane estimate $P^{l}={\left[\begin{array}{c} n^{l}\\ d^{l} \end{array}\right]}_{4\times 1}$ where $n^{l}\in R^{3}$ is the normal vector and $d^{l}\in R$ is the distance parameter, then this vector must satisfy the vector equality:

(1) $${\left[\begin{array}{cc} p_{1}^{T} & -1\\ p_{2}^{T} & -1\\ & \vdots \\ p_{N}^{T} & -1 \end{array}\right]}_{N\times 4}{\left[\begin{array}{c} n^{l}\\ d^{l} \end{array}\right]}_{4\times 1}=0,$$

subject to $\left|\right|n^{l}\left|\right|=1$. The actual computation can be performed by defining:

(2) $$A≐{\left[\begin{array}{c} p_{1}^{T}\\ p_{2}^{T}\\ \vdots \\ p_{N}^{T} \end{array}\right]}_{N\times 3},a≐{\left[\begin{array}{c} -1\\ -1\\ \vdots \\ -1 \end{array}\right]}_{N\times 1},x=n^{l},\;\mathrm{and}\;y=d^{l},$$

and replacing the equality by the minimization problem:

(3) $$\min_{x,y}\left|\right|\left[\begin{array}{cc} A & a \end{array}\right]\left[\begin{array}{c} x\\ y \end{array}\right]{\left|\right|}^{2}\;\mathrm{subject}\;\mathrm{to}\;||x||=1.$$

If the normalization equality would apply to the whole vector ${\left[\begin{array}{c} x\\ y \end{array}\right]}_{4\times 1},$ then the answer to this problem is immediate: the singular vector associated with the smallest singular value. The solution for the problem posed here is slightly more complex as seen in the next Lemma.

**Lemma** **1.** The optimal solution $\left(n^{*},d^{*}\right)$ to the constrained Least-Squares problem is such that

1. $n^{*}$ is the eigenvector for $\left(A^{T}A-\frac{A^{T}{\mathrm{aa}}^{T}A}{a^{T}a}\right)$ associated with the smallest eigenvalue, and
2. $d^{*}$ = $-\frac{1}{a^{T}a}a^{T}\mathrm{Ax}$.

**Proof.**  See Appendix B. ☐

In addition, some extra knowledge of the walls is used to help the refinement process. More specifically, only horizontal planes (e.g., floor, ceiling) and vertical planes (e.g., walls, planar obstacles) are considered during the plane extraction. Most buildings are comprised mainly of vertical and horizontal walls, thus the algorithm exploits this fact. Therefore, when refining the plane estimate, first the Z-component is checked to be approximately 0 or ±1. The corresponding normal’s Z-component is thereby set to 0, 1 or −1 accordingly and the normal is re-normalized. The best plane estimate, as described in [2], for a point cloud with *N* points is thus given by

(4) $$P^{*}=\underset{P^{l}}{\mathrm{argmax}}\sum_{i=1}^{N}\frac{1}{1+|n^{l}\cdot p_{i}-d^{l}|}.$$

In situations where walls in the floorplan are not vertical or horizontal (e.g., at a slant), those would simply not be included in the a priori map of wall information to utilize.

#### 4.3.3. Stopping Criterion

Because multiple walls could exist in a given point cloud, this algorithm continues to extract planes until it reaches a stopping criterion. Thus, a stopping criterion is required to know when the points left in the point cloud are noise and do not have any planar structure. To accomplish this, rather than relying on predefined thresholds, a classifier was trained via a Support Vector Machine (SVM) to classify a point cloud as containing a plane or not. The input to the classifier is the histogram $H_{e}$ for the best plane estimate, $P^{*}$.

**Figure 10:**
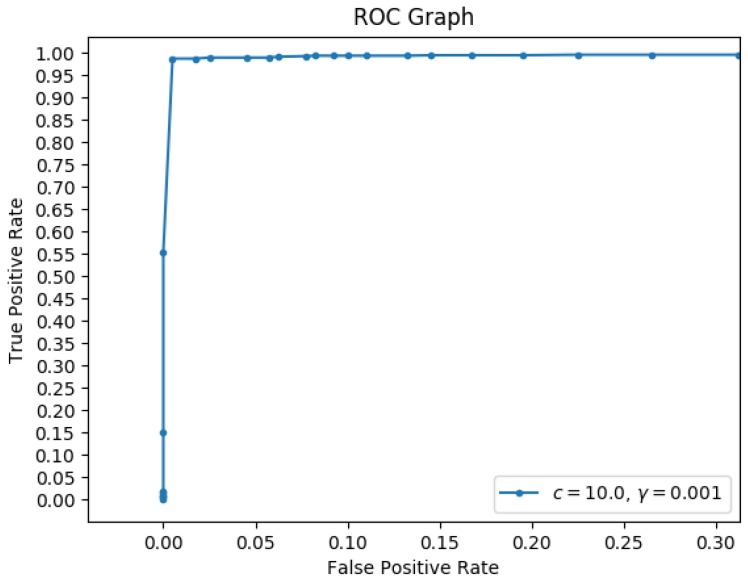
ROC graph of the SIPR stopping criterion classifier (different from that in [2]).

To train the SVM, synthetic data was used so that correct labels could be appropriately applied. Different synthetic data was used to train and test the SVM classifier than that presented in [2]. The number of planes in the environment varied from 0 to 5, the number of points on each plane from 50 to 2000, and the outlier percentage from 0 to 15%. In addition, 1400 environments were created and all of the histograms were exclusively separated into training data and testing data. One thousand histograms were used to train the SVM classifier and 1323 histograms were used to test it. Figure 10 is the Receiver Operating Characteristic (ROC) graph, showing approximately 0% false positive percentage and about 97% true positive percentage. Notice that, having extracted all of the planes in the scene, “corner points,” namely points that exist in the neighborhoods of wall intersections, require special attention. To do this, each point is compared with all of the computed planes and association is based on minimum plane-distance error. Although most points will remain corresponding to the plane which they created, some of them could belong to the adjacent wall.

### 4.4. Wall Plane Fusion

The *Wall Plane Fusion* algorithm focuses on forming associations between the extracted planes and the walls of the floorplan. In addition, because obstacles may be present in the environment, this algorithm handles removing them. In fact, in a scene, there are three types of obstacles which could exist:

1. Non-planar obstacles,
2. Planar obstacles whose normal vectors match some of the walls’ normals,
3. Planar obstacles whose normal vectors do not match any of the walls’ normals.

Examples of non-planar obstacles include chairs and tables, while types of planar obstacles include cabinets and bookshelves. It is assumed that there could exist planar obstacles which are right next to walls, but no obstacle entirely covers the wall so as to occlude the view of it. In the situation where an obstacle entirely covers a wall (e.g., shelving), then it is necessary that a different wall be in view. As shown later in this section, at least one wall needs to be in view to properly obtain the scale factor. Non-planar obstacles are removed during the SIPR algorithm because, as a result of extracting the planes, all planar *outliers* are removed. One of the goals of this algorithm then is identifying and eliminating *planar obstacles*.

#### 4.4.1. Computed Plane—Wall Relationship

As mentioned in [2], given some wall, W=(n,d) and some plane, $P=(\tilde{n},\tilde{d})$, defined by the normal vector and distance parameters ($n,\tilde{n}\in R^{3}$ and $d,\tilde{d}\in R$), the transformation between the plane and the wall is defined as follows:

(5) $$n=R\tilde{n},$$

(6) $$d=k\tilde{d}+t\cdot n,$$

for some rotation R∈SO(3), scale factor k∈R, and translation $t\in R^{3}$. The walls and planes are defined in the Hesse normal form. Each camera pose for the first batch of images in this initialization is transformed with respect to the camera at the first instance, so t refers to the camera position of the first camera instance.

It is important to note that the meaning of the distance parameter for walls is different than that for the computed planes. For the walls, *d* refers to the distance from the origin of the world coordinate system to the corresponding wall. For the computed planes, $\tilde{d}$ refers to the unscaled distance from the location of the first camera instance to the wall in view.

#### 4.4.2. Orientation Filter

**Figure 11:**
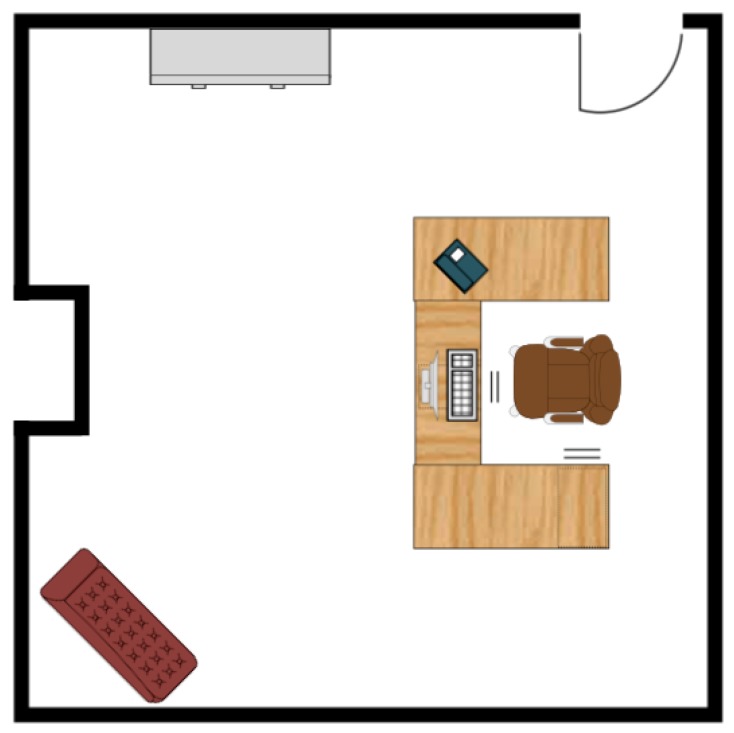
Example environment with planar obstacles and a wall protrusion.

The first stage of this algorithm consists of finding candidate walls whose normals align with those of the computed planes. Additionally, this part focuses on removing planar obstacles whose normals do not match any normals of walls (refer to the sofa in Figure 11). More specifically, given *N* walls and *M* computed planes, let WP be the set of N·M wall–plane pairs where $\mathrm{WP}=\{\left(W^{i},P^{j}\right)\}$. To remove such currently unseen walls and dissimilar planar obstacles, only wall–plane pairs which satisfy Equation (7) are kept, as discussed in [2]. Let $\left(W^{i},P^{j}\right)$ = $\left(n^{i},d^{i}\right),\left({\tilde{n}}^{j},{\tilde{d}}^{j}\right)$ be some wall–plane pair,

(7) $$e_{R}^{ij}<\delta_{R},$$

where

(8) $$e_{R}^{ij}=\left|1-n^{i}\cdot {\tilde{n}}^{j}\right|,$$

and $\delta_{R}$ is some threshold. For the experimentation done in this paper, $\delta_{R}$ was chosen to be 0.015. Thus, walls whose normals were different from any of the computed planes were removed as well as planar obstacles whose normals were different from the walls of the floorplan.

#### 4.4.3. Translation Filter

The next stage of the *Wall Plane Fusion* algorithm is to rule out cases of wall–plane pairs with matching normals but mismatching distance parameters. Example situations of this type are wall protrusions (refer to the wall protrusion in Figure 11) or planar obstacles with normals which match those of nearby walls (consider the file cabinet in Figure 11).

**Figure 12:**
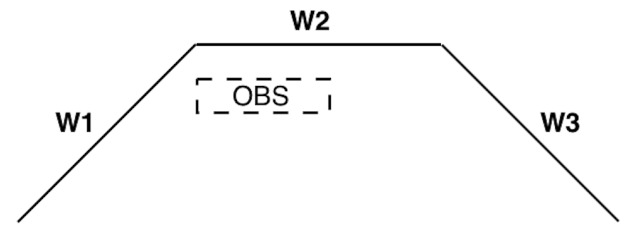
Situation with left and right concavity.

Consider the case where a wall has a normal vector which is unique to the current room. It follows that, if planes are found which share that normal, then, under specific concavity conditions, any planar obstacle(s) can be detected and the correct wall determined. More specifically, let W be the wall in view and $\left(P^{1},P^{2},\ldots \right)$ be the computed planes from the point cloud. Suppose that n, the normal of the wall, is unique to the current room in the building. Then, it follows (as shown in Figure 12) that if this particular wall (e.g., $W^{2}$) has left and right concavity, the correct plane to map to the wall is the one with the largest distance from the camera. Here, concavity is defined as the angle between two consecutive normals being acute from the perspective of the world coordinate system origin. The correct pair is $(W,P^{j}),$ where $d^{j}$ corresponds to the maximum distance from the camera for j=1,2,…

In other situations where left or right concavity does not occur, identification of obstacles is handled by evaluating pairwise characteristics of walls. In other words, because there is scale ambiguity present, the distance parameters cannot be compared directly; however, because the solution is correct relative to itself, it follows that relative characteristics between *pairs* of planes and *pairs* of walls are invariant to overall solution scaling. To handle this, the relationship between pairs of walls and pairs of planes is evaluated. We will denote such (wall-wall, plane-plane) pairs as “wall–plane collections” and define them as follows:

Let *L* be the number of filtered wall–plane pairs following the Orientation Filter. We define $C=\{\left(\left(W^{i},W^{j}\right),\left(P^{m},P^{l}\right)\right),\ldots \}$ to be the set of wall–plane collections with cardinality $\frac{L(L-1)}{2}$. Given some collection $\left(\left(W^{i},W^{j}\right),\left(P^{m},P^{l}\right)\right)$, it follows that if $W^{i}$ pairs with $P^{m}$ and $W^{j}$ pairs with $P^{l}$ in an ideal sense, the following equation holds (refer to [2]):

(9) $$\frac{d^{i}-t\cdot n^{i}}{d^{j}-t\cdot n^{j}}=\frac{k{\tilde{d}}^{m}}{k{\tilde{d}}^{l}}=\frac{{\tilde{d}}^{m}}{{\tilde{d}}^{l}}.$$

Therefore, for any plane pair which is not a direct match for the corresponding wall pair, whether that includes walls that should not be in view or planar obstacles, again in an ideal sense, Equation (9) does not hold. Thus, the translational error term for each collection is defined to be the following, as shown in [2]:

(10) $$e_{T}=\left|\frac{d^{i}-t\cdot n^{i}}{d^{j}-t\cdot n^{j}}-\frac{{\tilde{d}}^{m}}{{\tilde{d}}^{l}}\right|.$$

Note that $e_{T}$ is set to be such that $d^{i}-t\cdot n^{i}<d^{j}-t\cdot n^{j}$. In order to handle situations where similar geometry is present in various parts of the room, the encoder’s odometry information is introduced to provide appropriate weights. Thus, given a wall–plane collection, the individual scale factors are computed that would result from pairing corresponding walls and planes. These are denoted by $k_{1}$ and $k_{2}$. These scale factors are subsequently used to scale the translation vector between two Structure from Motion positions, and this is compared to the translation vector between the two corresponding encoder-formed positions. The weights are then appropriately used to find the best wall–plane collection.

The wheel encoders’ temporary position and orientation updates are governed by the following equations:

(11) $$\psi_{enc}^{s}=\psi_{enc}^{s-1}+\frac{r_{out}^{s}-r_{in}^{s}}{b},$$

(12) $$t_{enc}^{s}=t_{enc}^{s-1}+r^{s}\left[\begin{array}{c} \sin(\psi_{enc}^{s})\\ \cos(\psi_{enc}^{s})\\ 0 \end{array}\right],$$

where $r_{out}^{s}$ and $r_{in}^{s}$ are the distances traveled according to the outer and inner wheel encoder readings, respectively, $r^{s}$ is the average of $r_{out}^{s}$ and $r_{in}^{s}$, and *b* is the vehicle front axle baseline:

(13) $$k_{1}=\frac{d^{i}-t\cdot n^{i}}{{\tilde{d}}^{m}},$$

(14) $$k_{2}=\frac{d^{j}-t\cdot n^{j}}{{\tilde{d}}^{l}},$$

(15) $$\rho_{1}=\left|k_{1}\left(t_{sfm}^{s+1}-t_{sfm}^{s}\right)-\left(t_{enc}^{s+1}-t_{enc}^{s}\right)\right|,$$

(16) $$\rho_{2}=\left|k_{2}\left(t_{sfm}^{s+1}-t_{sfm}^{s}\right)-\left(t_{enc}^{s+1}-t_{enc}^{s}\right)\right|,$$

where $\rho_{1}$ and $\rho_{2}$ are the wall–plane collection weights. The best wall–plane collection is found by:

(17) $$C^{*}=\underset{\left(\left(W^{i},W^{j}\right),\left(P^{m},P^{l}\right)\right)}{\mathrm{argmax}}\left(\frac{1}{1+\min\left(\rho_{1},\rho_{2}\right)\cdot e_{T}}\right).$$

Given such a best wall–plane collection, the question is which pair is correct and if both are correct, which is better? To handle this, the scale factor that would be computed from each wall–plane pair is compared with the encoders’ rough scale factor, but that comparison is also weighted by the rotational error term. That way, in the case where both are correct, having the more accurate normal orientation can be taken into account in the selection process. Given any two camera translations returned from Structure from Motion, $t_{sfm}^{s}$ and $t_{sfm}^{s+1}$, the encoder rough scale estimate is computed as follows, as shown in [2]:

(18) $$k_{enc}=\frac{|t_{enc}^{s+1}-t_{enc}^{s}|}{|t_{sfm}^{s+1}-t_{sfm}^{s}|},$$

(19) $$\left(W^{*},P^{*}\right)=\underset{\left(W^{i},P^{m}\right)}{\mathrm{argmin}}\;\gamma_{im}\left|k_{enc}-\frac{d^{i}-t\cdot n^{i}}{{\tilde{d}}^{m}}\right|,$$

where $\gamma_{im}=e_{R}^{im}$. After applying this Translation Filter and obtaining the best wall–plane pair, the best scale estimate, $k^{*}$ is directly given, as stated in [2], by:

(20) $$k^{*}=\frac{d^{*}-t\cdot n^{*}}{{\tilde{d}}^{*}}.$$

The Structure from Motion relative camera positions and 3D points are subsequently scaled by $k^{*}$, and this best wall–plane pair is used as an external constraint for a constrained Bundle Adjustment optimization. Thus, it follows that a necessary condition for resolving the scale factor is that there exists only one wall–plane pair, corresponding to having only a single wall in view. Note that the focus of this work is to establish a positioning system with global scale. In this regard, the initial pose of the vehicle is known and multi-hypothesis localization is not considered.

## 5. Positioning Update Routine

After initialization, an update routine continues on to provide the global positioning. Structure from Motion is used to obtain the new camera pose and the new 3D points. The new camera pose and 3D points are subsequently transformed to be in the same coordinate frame as the previous batch of images and then scaled by $k^{*}$. After scaling, the 3D points are mapped to walls.

### Point-Wall Mapping

For all subsequent localization, it is necessary to determine whether the 3D points lie on the walls of the floorplan or whether they are outliers. Therefore, the following defines the mapping between each point $p_{i}$ and a potential corresponding wall W from among the walls of the floorplan, $W^{l}=\left(n^{l},d^{l}\right)$.

For each point $p_{i}$,

(21) $$\left(W,e_{i}\right)=\underset{W^{l}}{\mathrm{argmin}}\left|p_{i}\cdot n^{l}-d^{l}\right|.$$

The point $p_{i}$ is then mapped to wall $W_{i}$ such that

(22) $$W_{i}=\left\{\begin{array}{cc} W, & \mathrm{if}\;e_{i}<\tau ,\\ \emptyset , & \mathrm{otherwise}. \end{array}\right.$$

Here, τ is some threshold, and, for the experimentation in this paper, it was chosen to be 15 cm. In other words, inlier 3D points were tolerated up to 15 cm off of their corresponding wall before refinement in this update routine. The best wall was selected to be the one which had the most inliers.

## 6. Solution Refinement—Wall Constrained Bundle Adjustment

After computing an initialization using the algorithm described in Section 4 or an update solution using the algorithm in Section 5, a Wall Constrained Bundle Adjustment is performed to refine the solution at global scale. Thus, this algorithm is used to improve the initial solution and to improve the update solution. First, the cost function contains a reprojection error term given by:

(23) $$E=\sum_{i,j}\kappa_{ij}\left|\right|{\tilde{p}}_{i}^{j}-{\hat{p}}_{i}^{j}{\left|\right|}^{2},$$

where ${\tilde{p}}_{i}^{j}$ represents the *i*th reprojected 3D point seen from the camera at the *j*th instance and ${\hat{p}}_{i}^{j}$ corresponds to the *i*th observed 2D point seen in the *j*th frame. In addition, $\kappa_{ij}$ is a weight that is inversely proportional to the distance each 3D point is from each camera.

Here,

(24) $${\tilde{p}}_{i}^{j}={\left[{\left(v_{i}^{j}\right)}_{x}/{\left(v_{i}^{j}\right)}_{z},{\left(v_{i}^{j}\right)}_{y}/{\left(v_{i}^{j}\right)}_{z}\right]}^{T}\mathrm{where}\;v_{i}^{j}=KR\left(\Psi^{j}\right)\left(p_{i}-t^{j}\right),$$

where $K\in R^{3\times 3}$ is the intrinsic camera calibration matrix, $\left(T^{j},\Psi^{j}\right)$ is the camera pose, and $p_{i}$ is the 3D world point. In addition, $\kappa_{ij}={\left(\frac{{\left(v_{i}^{j}\right)}_{z}}{f}\right)}^{2},$ where *f* is the focal length of the camera in pixels.

Let ${\mathrm{WP}}^{*}=\left(W^{*},P^{*}\right)$ be the best wall–plane pair found from *Wall Plane Fusion* where $W^{*}=\left(n^{*},d^{*}\right)$. Furthermore, let $p_{m}\in R^{3}$ denote all of the world points which are supposed to lie on the wall $W^{*}$. In an ideal sense, if the points $p_{m}$ lie exactly on the wall, then it follows that:

(25) $$p_{m}\cdot n^{*}-d^{*}=0.$$

Therefore, a soft constraint is introduced thereby allowing feature points which are not exactly on the wall to be tolerated in a more robust way:

(26) $$\kappa_{w}\sum_{m}{\left(p_{m}\cdot n^{*}-d^{*}\right)}^{2},$$

where $\kappa_{w}$ is a tuned weight. In addition, there are other inherent platform constraints which can be applied. Note that the camera is rigidly mounted onto the deck of the vehicle at a known height, $h_{c}$. It follows that the camera remains on average at a roughly constant altitude throughout execution. Therefore, a soft constraint, as also shown in [2], is appended to handle any natural vertical movement that may occur. Note that the vehicle contains shocks:

(27) $$\kappa_{c}\sum_{j}{\left({\left(t^{j}\right)}_{Z}-h_{c}\right)}^{2},$$

with ${\left(t^{j}\right)}_{Z}$ corresponding to the Z-component (in world coordinates) of the camera at the *j*th instance and $\kappa_{c}$ serves as a weight. Furthermore, in the same regard, the roll and pitch angles of the camera on average are roughly constant and close to 0. Therefore, the cost function is augmented to incorporate this knowledge in the form of two more soft constraints

(28) $$\kappa_{a}\sum_{j}{\left(\phi^{j}-0\right)}^{2},$$

(29) $$\kappa_{a}\sum_{j}{\left(\theta^{j}-0\right)}^{2},$$

with weight $\kappa_{a}$. The Bundle Adjustment weights were chosen through tuning, and higher weight was placed on the orientation cost terms compared to that of the world points or camera height. For the experimentation done in this paper, $\kappa_{w}=1,\kappa_{c}=1,$ and $\kappa_{a}=500$. Thus, the entire modified cost function is:

(30) $$E=\sum_{i,j}\kappa_{ij}\left|\right|{\tilde{p}}_{i}^{j}-{\hat{p}}_{i}^{j}{\left|\right|}^{2}+\kappa_{w}\sum_{m}{\left(p_{m}\cdot n^{*}-d^{*}\right)}^{2}+\kappa_{c}\sum_{j}{\left({\left(t^{j}\right)}_{Z}-h_{c}\right)}^{2}+\kappa_{a}\sum_{j}{\left(\phi^{j}-0\right)}^{2}+\kappa_{a}\sum_{j}{\left(\theta^{j}-0\right)}^{2}.$$

Note that, while there may be multiple wall–plane associations, only *one* wall–plane pair is necessary to resolve the scale. Using only the best wall–plane pair reduces the computational cost by only optimizing points on this computed plane. As a result, global positioning is achieved.

## 7. Positioning System Computational Runtime

This section contains a discussion of the current and future expected run-times of the positioning algorithm. Instead of attempting to establish actual complexity, a general indication is provided, pointing to the most time-consuming aspects and their proposed solutions.

First, note that there is a wide difference between the initialization stage and the continuous operation. Since it is assumed here that the former is performed only once, attention was restricted to the latter. Specifically, the main efforts were placed in making the update routine as efficient as possible. Currently, the constrained Bundle Adjustment and the RANSAC module dominate the complexity of the overall scheme with the former sometimes taking an order of magnitude more time than the latter. Other computations did not significantly affect the computation times. For example, for data from one of the simulation tests presented later in Section 8, matching features between two frames during the update routine took 2.02 s and the constrained Bundle Adjustment optimization took 13.23 s. The overall goal is to achieve near real-time behavior, since one can rely on the other sensors available on the vehicle (e.g., encoder odometry and inertial navigation using IMU readings) to keep a navigation solution updating in hard real time. In order to meet this objective, work is in progress in two directions:

1. Bundle Adjustment (BA). An efficient implementation of the BA has been proposed in the literature and one of them was implemented and tested. It was observed that the additional wall constraints heavily affect computational times and hence alternative routes are currently being explored. For example, because the camera height and camera roll and pitch angles are known, then after the update routine computes a solution for the most recent camera, rather than using the computed values for the height and roll and pitch angles, they can be set directly to their known values. In addition, rather than setting the parameter τ described in Section 5 to be an inlier constant, τ can be set dynamically using the previous batch’s minimal point error. These were done in the second set of the simulation experiments presented, as mentioned later in the paper.
2. RANSAC. The RANSAC algorithm is known to be capable of extracting a large number of features while being computationally expensive. Alternative classifiers are currently being considered that will give satisfactory performance under the constraints of the problem at hand. For example, more specialized filtering can be done on the image correspondences to reduce the required number of iterations.

## 8. Testing

To validate the system, extensive testing was performed on synthetic and simulated scenarios, and then an actual lab test was conducted to verify the synthetic and simulated results. Note that synthetic testing did not use images, but rather image points were synthesized by generating world points and projecting them according to a pre-defined camera calibration matrix. Simulated testing was accomplished via *Unreal Engine* and *Microsoft Airsim*. Realistic simulated images of a built indoor environment were used, and this is especially a benefit to this paper as indoor positioning testing in simulated environments is not very prevalent in current literature.

### 8.1. Synthetic Testing

**Figure 13:**
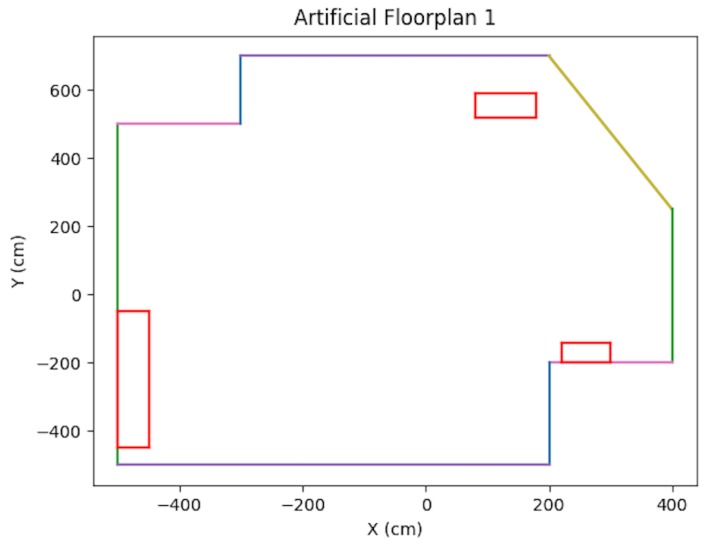
Artificial Floorplan 1, see [2].

**Figure 14:**
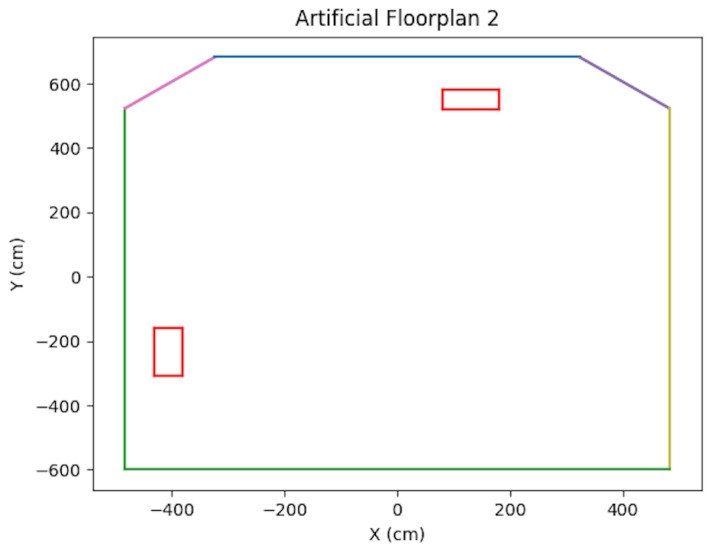
Artificial Floorplan 2, see [2].

In the synthetic case, artificial floorplans were created containing obstacles. Refer to Figure 13 and Figure 14. For each scenario, a vehicle trajectory was created in a loop shape with curves and bends so that the vehicle could view the majority of the walls. Artificial tracks were created for the 3D points which were located on walls, obstacles, and some on the floor. Points placed on obstacles were verified via ray tracing. Additional synthetic testing parameters are presented in Table 1.

**Figure 15:**
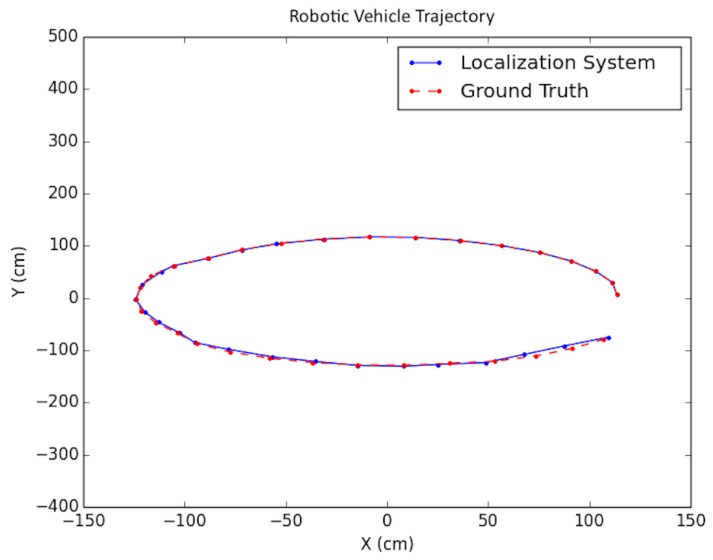
Synthetic test trajectory 1 (different from that in [2]).

**Figure 16:**
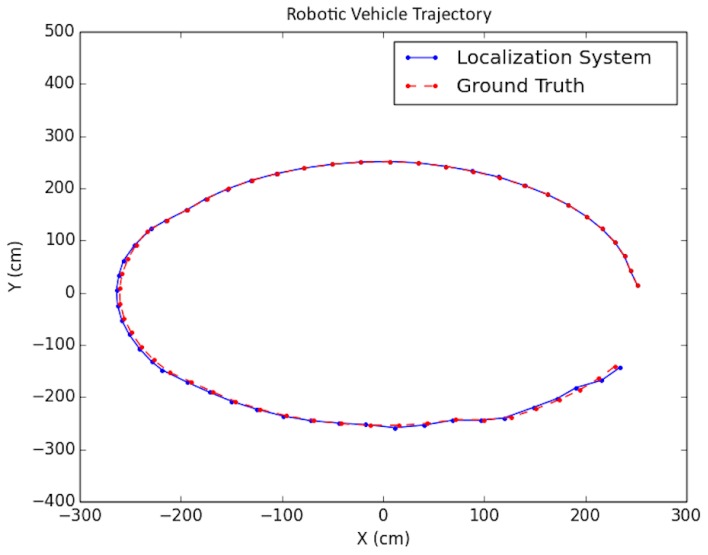
Synthetic test trajectory 2 (different from that in [2]).

As shown in the table, in order to simulate noise on image points, half-pixel noise was applied to each of the points after projecting them onto the image plane. The camera calibration matrix used to project the generated 3D points onto the artificial image plane was taken to be the same as the physical camera used in the real world experimentation in Section 8.3. In addition, to simulate encoder odometry, 7% error was applied to the waypoint positions of the cameras. This value was chosen based on real-data experimentation. A sample trajectory for each artificial floorplan is shown in Figure 15 and Figure 16. The results for both types of artificial floorplans are presented in Table 2. Ten runs were performed for each type of artificial floorplan. The results were obtained as follows: For each run, the error between each solution position and ground truth position was obtained and then averaged for all positions in the trajectory. The average error was subsequently averaged across all runs. The standard deviation of the position error was obtained in a similar manner as well as the orientation counterparts. Furthermore, this computation of the error was used consistently throughout the experimentation results analyses.

### 8.2. Microsoft Airsim Simulation Testing

**Figure 17:**
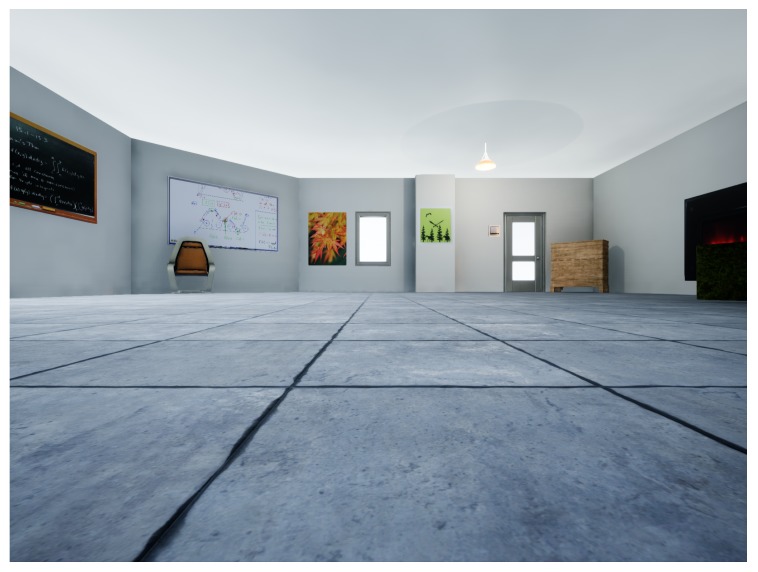
Simulation indoor environment.

**Figure 18:**
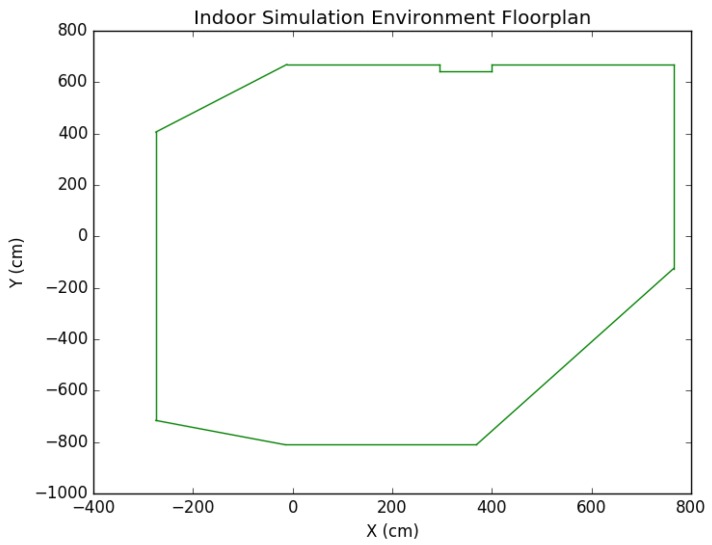
Indoor simulation environment floorplan.

To further test the positioning system, we built an indoor simulation environment using *Unreal Engine* and *Microsoft Airsim* (see Figure 17 for a sample view). Obstacles such as a chair, a sofa, a cabinet, and a bookshelf were placed in the environment to provide both non-planar and planar obstacles. The scene also contained other standard features such as a door, windows, a fireplace, and more. In addition, a wall protrusion was included so that the environment contained quite a bit of complexities. In fact, the simulated environment was designed to be comparable to the real experiment environment if not by detail then at least by general constraints it imposes on the localization scheme. For instance, the number of walls, the wall protrusion, and similar obstacles were designed to create an environment similar to the real lab setting. A single, simulated environment was used for all experimentation, and the number of features detected during computation was about 3–4 times as many as those in the real world. The floorplan is shown in Figure 18.

**Figure 19:**
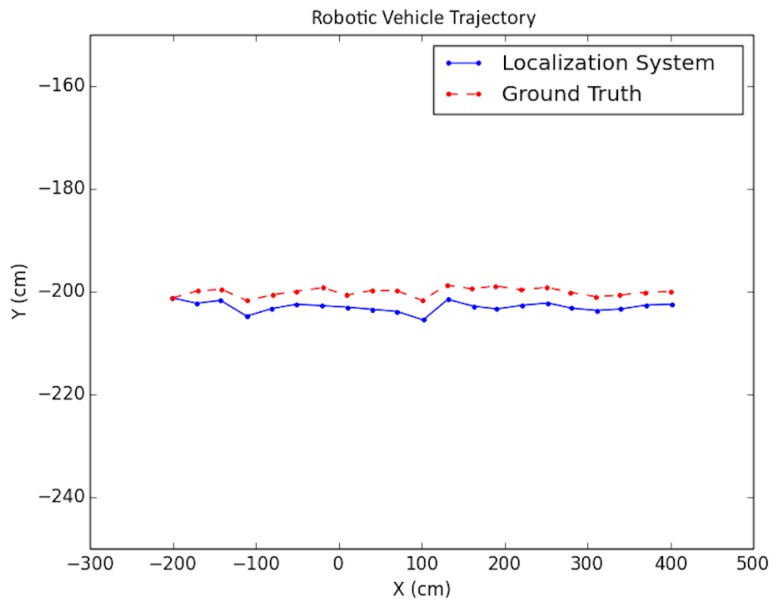
Simulation test trajectory 1.

**Figure 20:**
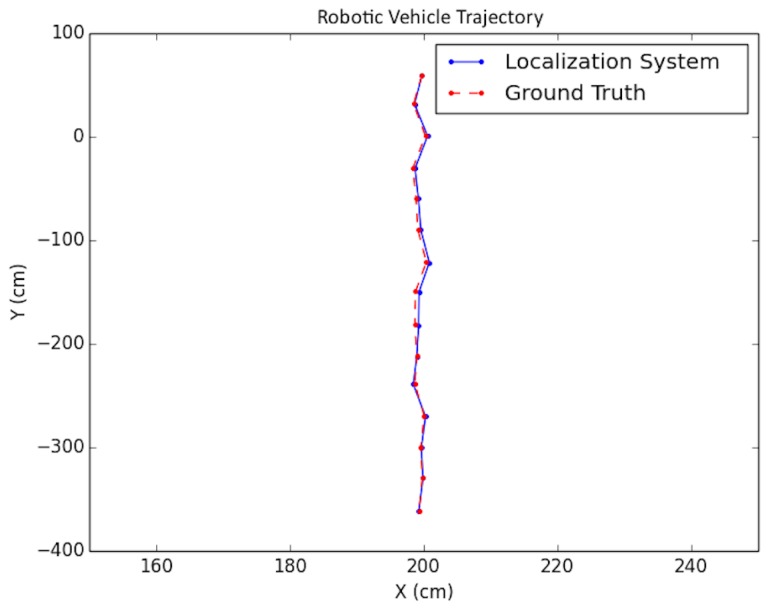
Simulation test trajectory 2.

**Figure 21:**
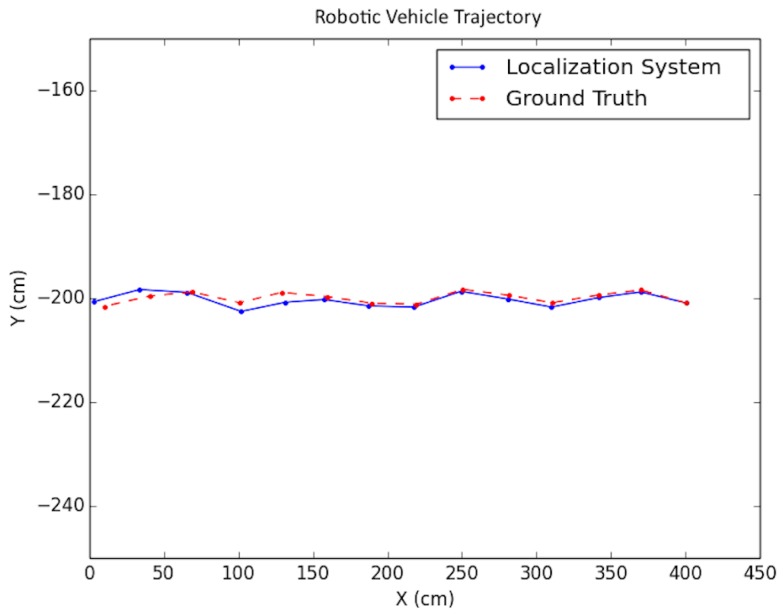
Simulation test trajectory 3.

Each experiment was performed as follows: A trajectory for the car moving in the scene was defined; next, images from each viewpoint were rendered from the simulated environment and captured using the functionality provided by *Microsoft Airsim* with a similar field of view and altitude as in the experimental testing discussed in the next subsection. To simulate encoder odometry, encoder tick readings were artificially produced where 1% systematic error (the vehicle front axle length and wheel radius) was applied, non-systematic error was introduced according to [24], and quantization was applied to the artificial encoder readings. Finally, the images, together with an initial pose provided for initialization and wheel encoder readings, were fed as input together into the positioning system. Three types of trajectories were created viewing different parts of the environment with sample tests shown in Figure 19, Figure 20 and Figure 21. For each type, the position of the vehicle was randomized, namely the position of the camera where the images were taken. In total, ten randomized variations were run for each of the trajectory types and the result statistics are provided in Table 3.

The average values due to a limited number of tests were not large enough to rule out the hypothesis that the mean error was 0. The average standard deviation of the error was about 1/10 the width of the car, so this value seems to be good enough for most applications.

**Figure 22:**
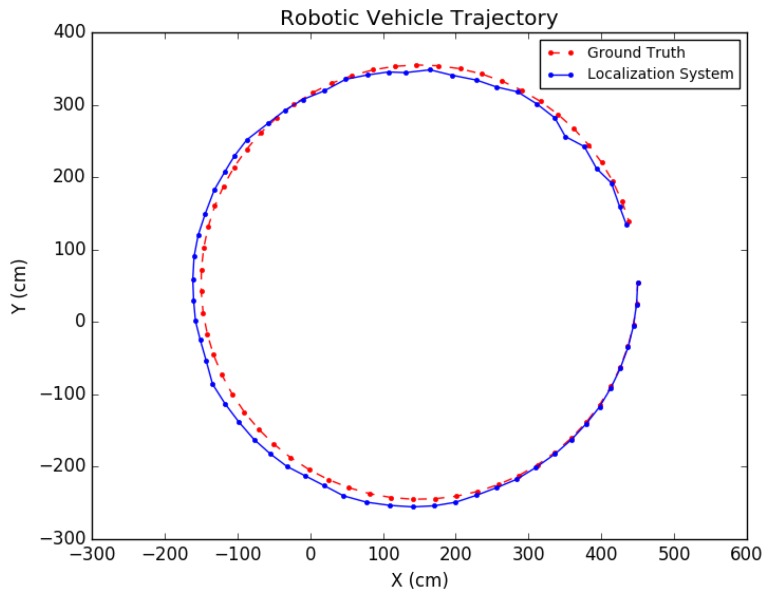
Simulation test trajectory 4.

**Figure 23:**
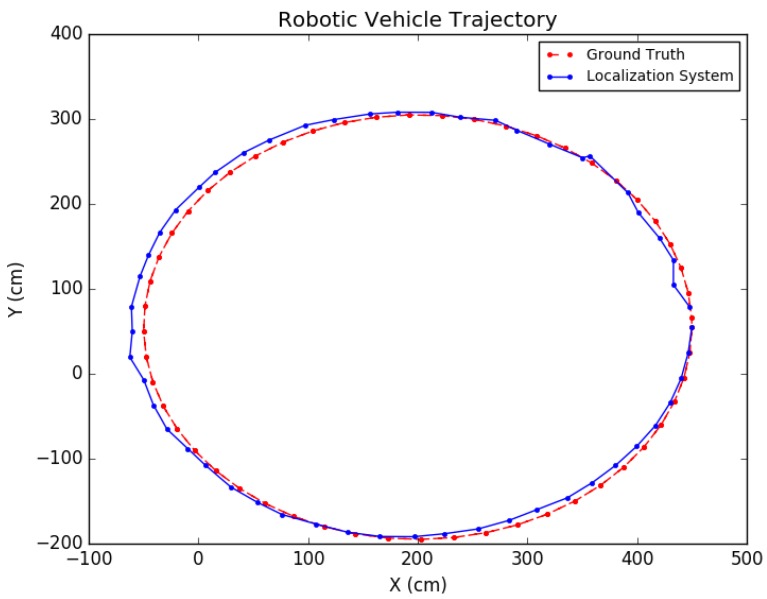
Simulation test trajectory 5.

The proposed positioning system was further tested with longer trajectories by navigating around the indoor simulation environment in a circular fashion, viewing the entire environment. In these experiments, as mentioned in Section 7, after computing the pose of the camera in the update routine, the known values of the camera height and roll and pitch angles were set directly for that pose. In addition, the inlier error parameter τ described in Section 5 was dynamically set each iteration, using a weighted average between the previous batch’s minimal point error and the current batch’s minimal point error. Note that minimal point error corresponds to the minimum distance a point is from the wall on which it is supposed to be. More specifically, if $\epsilon_{i}$ represents the *i*th batch’s minimal point error, then $\tau_{i}=0.6\epsilon_{i-1}+0.4\epsilon_{i}$. The results for these tests are shown in Table 4 and the trajectories are presented in Figure 22 and Figure 23.

### 8.3. Real Scenario Testing

**Figure 24:**
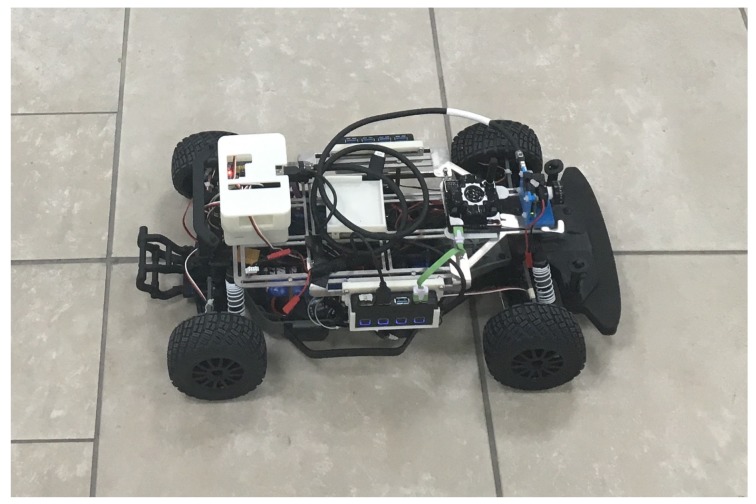
Robotic vehicle, see [2].

**Figure 25:**
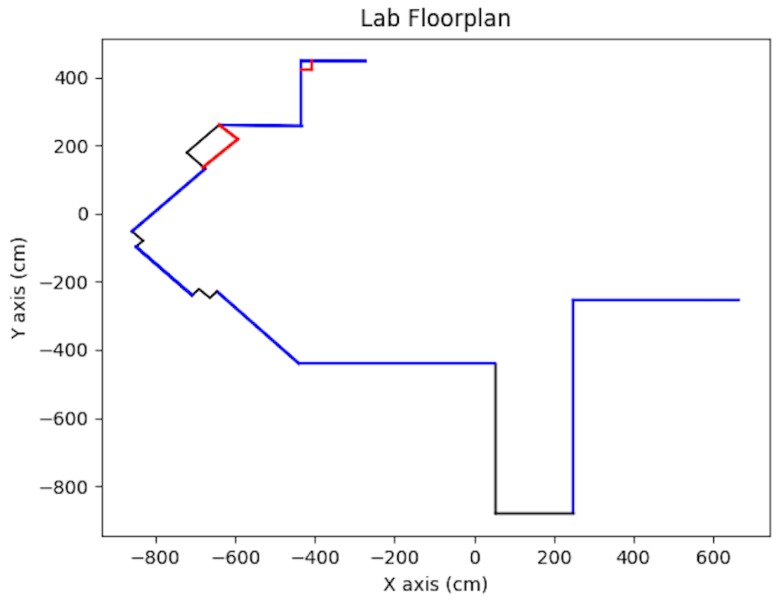
Lab floorplan, see [2].

In addition to synthetic tests and simulation tests, real scenario testing was done. The test vehicle was the second generation BARC platform augmented with a monocular camera (IDS Imaging Development Systems GmbH, Obersulm, Germany) [25] mounted onto the deck of the vehicle at a height of 14.7 cm (refer to Figure 24). Extensive details on the BARC vehicle can be found at [26]. The on-board camera provided up to 57 fps at full resolution of 3.2 MP with a field of view of 65.6 × 51.6 degrees. The vehicle length was 53.5 cm and its width was 28.1 cm. For ground-truth, data which combined both manual and image-based measurements was used. The lab floorplan is shown in Figure 25 with active walls shown in blue. The images were captured by the computer on the vehicle and having the vehicle work autonomously is expected to happen in the near future. At the current stage, the vehicle was used for data acquisition and actual computations were done offline on a PC computer.

**Figure 26:**
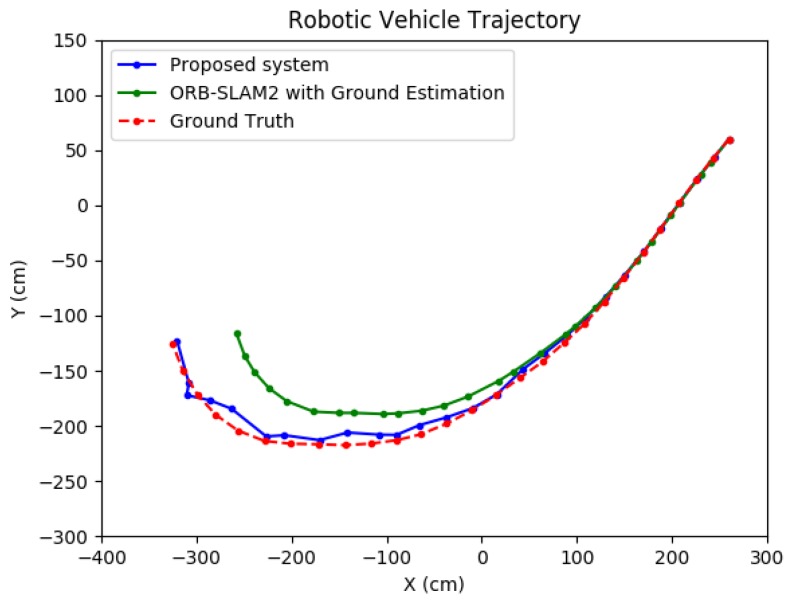
Real-world test comparison.

For one such test, the proposed method was compared to a method which coupled ORB-SLAM2 [4] with a ground plane estimation algorithm. Because ORB-SLAM2 does not provide *global scale* camera motion automatically, the scale was obtained during an initialization framework which ran LIBVISO2 [27] on the first nine frames to extract the scale from ground plane estimation using the camera height. This scale value was subsequently applied to the entire ORB-SLAM2 solution. The results are presented in Table 5. The proposed system performed with significantly higher accuracy in both position and orientation. As shown in Figure 26, due to the undershoot nature of the ORB-SLAM2 trajectory, it can be seen that computing the global scale using a wall–plane pair with the proposed system was more effective than relying on ground plane estimation. Thus, as can be seen, the standard deviation of the positioning error for the proposed system was shown to be about five times less than that for the compared system in the *x*-direction. In addition, the standard deviation of the orientation error of the proposed system was about 2.5 times less than that of the ORB-SLAM2 system. Because the scale factor does not directly affect the orientation, this result is attributed to the refinement from the constrained Bundle Adjustment optimization which utilizes a wall–plane pair as an external constraint.

## 9. Conclusions

This work proposes a vision-based indoor positioning system of a small robotic vehicle. In comparison to contemporary approaches, the proposed system achieves global positioning without any dense 3D map or prior scene exploration or infrastructure installation. By utilizing the sparse knowledge of the floorplan and providing special algorithms to associate extracted planar structure with a wall of the building, the scale factor, which is inherently lacking in monocular vision, is able to be resolved. A specially designed Bundle Adjustment optimization provides refinement for the camera motion while enforcing the localization solution to be at global scale. It was shown that, in order to obtain global positioning, only a single wall needs to be in view. Throughout this paper, an idealized floorplan with no errors was assumed. The setup of the problem was idealized also in other senses such as no illumination problems were assumed and the camera was assumed to be perfectly calibrated. The effect of more realistic assumptions on the performance of the algorithm is currently under study together with other possible sources of localization error. 

## Figures and Tables

**Table 1:** Synthetic testing parameters.

Parameter	Value
Number of Frames	30, 51 (type 1, type 2, resp.)
Waypoint Baseline (cm)	20
Image Noise (px)	0.5
Encoder Odometry Error (%)	7

**Table 2:** Synthetic testing results.

Artificial Floorplan Type	Avg. Traj. Length (cm)	${\bar{e}}_{\mathrm{pos}}$ (cm)	${\bar{\sigma }}_{\mathrm{pos}}$ (cm)	${\bar{e}}_{\mathrm{yaw}}$ (rad)	${\bar{\sigma }}_{\mathrm{yaw}}$ (rad)
Type 1	659	(1.41, 0.06)	(3.93, 4.16)	0.0010	0.0073
Type 2	1382	(0.72, 0.36)	(0.72, 1.14)	0.0015	0.0040

**Table 3:** Advanced simulation results set 1.

Trajectory Type	Traj. Length (cm)	${\bar{e}}_{\mathrm{pos}}$ (cm)	${\bar{\sigma }}_{\mathrm{pos}}$ (cm)	${\bar{e}}_{\mathrm{yaw}}$ (rad)	${\bar{\sigma }}_{\mathrm{yaw}}$ (rad)
Type 1	600	(−0.59, 0.11)	(3.06, 4.06)	0.00076	0.0043
Type 2	420	(−0.15, −0.70)	(0.72, 1.14)	0.0015	0.0012
Type 3	390	(0.90, 2.19)	(3.00, 2.68)	0.0043	0.0096

**Table 4:** Advanced simulation results set 2.

Trajectory Type	Traj. Length (cm)	${\bar{e}}_{\mathrm{pos}}$ (cm)	${\bar{\sigma }}_{\mathrm{pos}}$ (cm)	${\bar{e}}_{\mathrm{yaw}}$ (rad)	${\bar{\sigma }}_{\mathrm{yaw}}$ (rad)
Type 4	1854.95	(6.75, 7.53)	(5.11, 3.97)	0.014	0.0076
Type 5	1560.0	(8.99, −3.81)	(4.05, 4.64)	0.016	0.027

**Table 5:** Real-world test comparison.

Method	Approx. Traj. Length (cm)	${\bar{e}}_{\mathrm{pos}}$ (cm)	${\bar{\sigma }}_{\mathrm{pos}}$ (cm)	${\bar{e}}_{\mathrm{yaw}}$ (rad)	${\bar{\sigma }}_{\mathrm{yaw}}$ (rad)
Proposed	728	**(−0.39, −4.49)**	**(4.09, 5.46)**	**−0.0022**	**0.034**
ORB-SLAM2 + Ground Plane Est.	728	(−29.16, −18.86)	(20.03, 8.87)	0.016	0.087

## Appendix A. Probabilities for the Initial Plane Computation

Let $S_{1}$, $S_{2}$, and $S_{3}$ be the three subsets of the point cloud. Furthermore, let $p_{1},p_{2},p_{3}$ be the three points selected to form the plane (refer to [2]):

(A1) $$\mathrm{Pr}\left(p_{1}\in S_{i}\right)=\frac{1}{3},$$

(A2) $$\mathrm{Pr}(p_{2}\in S_{i}|p_{1}\in S_{i})=\alpha ,$$

(A3) $$\mathrm{Pr}\left(p_{2}\in S_{j}|p_{1}\in S_{i}\right)=\left\{\begin{array}{cc} 1-\alpha , & \mathrm{if}\;j\neq i\;\mathrm{and}\;S_{i}\;\mathrm{adj}\;\mathrm{only}\;\mathrm{to}\;S_{j},\\ \frac{1-\alpha }{2}, & \mathrm{if}\;j\neq i\;\mathrm{and}\;S_{i}\;\mathrm{adj}\;\mathrm{to}\;S_{j}\;\mathrm{and}\;\mathrm{another}\;\mathrm{subset},\\ 0, & \mathrm{otherwise}, \end{array}\right.$$

(A4) $$\mathrm{Pr}\left(p_{3}\in S_{i}|p_{2}\in S_{i}\wedge p_{1}\in S_{i}\right)=\beta \;\mathrm{if}\;S_{i}\;\mathrm{adj}\;\mathrm{to}\;\mathrm{only}\;\mathrm{one}\;\mathrm{subset},$$

(A5) $$\mathrm{Pr}\left(p_{3}\in S_{j}|p_{2}\in S_{i}\wedge p_{1}\in S_{i}\right)=\left\{\begin{array}{cc} 1-\beta & \mathrm{if}\;j\neq i\;\mathrm{and}\;S_{i}\;\mathrm{adj}\;\mathrm{only}\;\mathrm{to}\;S_{j},\\ \frac{1-\beta }{2} & \mathrm{if}\;j\neq i\;\mathrm{and}\;S_{i}\;\mathrm{adj}\;\mathrm{to}\;S_{j}\;\mathrm{and}\;\mathrm{another}\;\mathrm{subset}, \end{array}\right.$$

(A6) $$\mathrm{Pr}\left(p_{3}\in S_{j}|p_{2}\in S_{i}\wedge p_{1}\in S_{j}\right)=\frac{1}{2}\;\mathrm{if}\;j\neq i\;\mathrm{and}\;S_{i}\;\mathrm{adj}\;\mathrm{to}\;S_{j}.$$

Note that α and β are tuning parameters and β>α. For the experimentation presented in this paper, α was chosen to be 0.6 and β was selected to be 0.7.

## Appendix B. Proof of the Planar Extraction Lemma

Introducing Lagrange multipliers:

(A7) $$L\left(x,y,\lambda \right)=\frac{1}{2}\left[\begin{array}{cc} x^{T} & y \end{array}\right]\left[\begin{array}{cc} A^{T}A & A^{T}a\\ a^{T}A & a^{T}a \end{array}\right]\left[\begin{array}{c} x\\ y \end{array}\right]+\frac{1}{2}\lambda \left(1-x^{T}x\right),$$

(A8) $$L^{\prime }\left(x,y,\lambda \right)=\left[\begin{array}{cc} A^{T}A & A^{T}a\\ a^{T}A & a^{T}a \end{array}\right]\left[\begin{array}{c} x\\ y \end{array}\right]-\left[\begin{array}{c} \lambda x\\ 0 \end{array}\right]=\left[\begin{array}{c} 0\\ 0 \end{array}\right],$$

From the second row:

(A9) $$a^{T}Ax+a^{T}ay=0,$$

(A10) $$y=-\frac{1}{a^{T}a}a^{T}Ax,$$

From the first row:

(A11) $$A^{T}Ax+A^{T}ay-\lambda x=0,$$

Plugging in *y* gives:

(A12) $$A^{T}Ax-\frac{A^{T}aa^{T}A}{a^{T}a}x-\lambda x=0,$$

(A13) $$\left(A^{T}A-\frac{A^{T}aa^{T}A}{a^{T}a}\right)x=\lambda x.$$

To find *x*, the above eigenvalue problem is solved. Furthermore, the optimal solution is one such that λ is minimized:

(A14) $$\begin{array}{cc} \left|\right|\left[\begin{array}{cc} A & a \end{array}\right]\left[\begin{array}{c} x\\ y \end{array}\right]{\left|\right|}^{2} & =\left[\begin{array}{cc} x^{T} & y \end{array}\right]\left[\begin{array}{cc} A^{T}A & A^{T}a\\ a^{T}A & a^{T}a \end{array}\right]\left[\begin{array}{c} x\\ y \end{array}\right]\\ & =\left[\begin{array}{cc} x^{T} & y \end{array}\right]\left[\begin{array}{c} \lambda x\\ 0 \end{array}\right]\\ & =\lambda x^{T}x\\ & =\lambda . \end{array}$$

Thus, minimizing $\left|\right|\left[\begin{array}{cc} A & a \end{array}\right]\left[\begin{array}{c} x\\ y \end{array}\right]{\left|\right|}^{2}$ corresponds to minimizing λ.
